# Predicting mood swings in women of reproductive age using machine learning on metabolic, menstrual, and lifestyle indicators

**DOI:** 10.3389/fgwh.2025.1700324

**Published:** 2026-01-02

**Authors:** Rawan AlSaad, Farah El Rayess, Rajat Thomas

**Affiliations:** 1 Weill Cornell Medicine-Qatar, Doha, Qatar; 2 Women’s Wellness and Research Center, Hamad Medical Corporation, Doha, Qatar

**Keywords:** artificial intelligence, machine learning, lifestyle, metabolic, women's health, mental health

## Abstract

**Background:**

Mood swings in reproductive-age women arise from interacting hormonal, metabolic, and lifestyle factors, yet scalable screening tools remain limited. Artificial intelligence (AI) and machine learning (ML) approaches offer the potential to integrate diverse predictors and enable early, data-driven risk stratification.

**Objective:**

To evaluate the performance of ML algorithms in predicting mood swings among reproductive-age women using menstrual, metabolic, and lifestyle survey data and to identify the most influential predictors.

**Methods:**

The study cohort included 465 reproductive-age women, with fifteen survey-derived features categorized into metabolic (e.g., BMI, recent weight gain, polycystic ovary syndrome), menstrual (regular periods, period length), lifestyle (fast-food consumption, daily exercise), symptom burden score, and demographic (age) categories. We compared five ML models: Random Forest, SVM, Gradient Boosting, LightGBM, and CatBoost, using precision, recall, F1, accuracy, and AUCPR metrics. Feature importance was assessed with permutation feature importance (PFI) and shapley additive explanations (SHAP).

**Results:**

Across models, the highest values achieved were precision 0.83, recall 0.91, accuracy 0.74, and AUCPR 0.87. PFI and SHAP converged on symptom burden as the dominant predictor, with additional signal from lifestyle indicators (higher fast-food consumption, lower daily exercise) and metabolic/dermatologic markers. Menstrual regularity/length contributed minimally; age showed a modest inverse association.

**Conclusions:**

Low-cost, self-reported features can support ML prediction of mood swings in reproductive-age women with good performance. Findings motivate prospective validation, dynamic prediction with wearables, and evaluation of AI-based approaches for early detection of women's mental health concerns in community and primary care settings.

## 1 Introduction

Mood swings refer to rapid, often intense fluctuations in emotional states, such as sudden shifts between feelings of happiness, irritability, sadness, or anxiety (1). While mood variability is a normal part of emotional life, especially in response to stress or hormonal changes, excessive or frequent mood swings can impair daily functioning and may signal underlying physiological or psychological conditions (2–5). In women of reproductive age, mood swings are commonly associated with hormonal fluctuations linked to the menstrual cycle, but they may also reflect broader lifestyle, metabolic, or mental health factors (6, 7). Although not classified as a standalone clinical disorder, mood swings are recognized as a prominent symptom in conditions such as premenstrual dysphoric disorder, polycystic ovary syndrome (PCOS), depression, and anxiety.

A growing body of literature indicates that reproductive, metabolic, and lifestyle factors jointly shape mood trajectories in reproductive-age women. Fluctuations in estrogen and progesterone across the menstrual cycle, particularly in the late luteal phase, have been linked to irritability, low mood, and heightened emotional reactivity, and may underpin premenstrual syndrome and premenstrual dysphoric disorder in susceptible women (8–11). In parallel, metabolic conditions such as obesity and polycystic ovary syndrome (PCOS) are consistently associated with higher rates of depressive and anxiety symptoms, potentially mediated by insulin resistance, hyperandrogenism, and chronic low-grade inflammation (12–14). Lifestyle behaviors further modify risk: observational studies link greater fast-food or energy-dense dietary intake and lower physical activity with more severe depressive symptoms and poorer emotional well-being in women (15–19). Collectively, these findings support an interlinked reproductive–metabolic–lifestyle axis contributing to mood instability, yet few studies have simultaneously modeled these domains to quantify their relative contributions to mood swings in everyday settings.

Recent advances in artificial intelligence (AI) and machine learning (ML) have demonstrated promise in predicting a range of mental health outcomes using low-cost and easily accessible data sources (20, 21). In this context, patient-reported survey data offer a valuable resource to model emotional health indicators. ML algorithms are particularly well-suited to identify complex, non-linear patterns in high-dimensional datasets, and have been increasingly applied in personalized healthcare.

Several studies have applied AI, ML, and wearable devices to stress-related outcomes in women across reproductive stages. Sharma et al. (22) developed a deep recurrent neural network achieving >95% accuracy in detecting stress among working women, while Hurwitz et al. (23) used Fitbit-derived biomarkers to identify postpartum depression. Ng et al. (24) combined wearable ECG with smartphone surveys to predict daily stress in pregnant women, and Zafar et al. (25) built hybrid RNN-LSTM models for perinatal depression screening. Together, these studies highlight the growing role of AI-driven approaches and digital health technologies for stress detection and monitoring in women's health.

**Figure 1:**
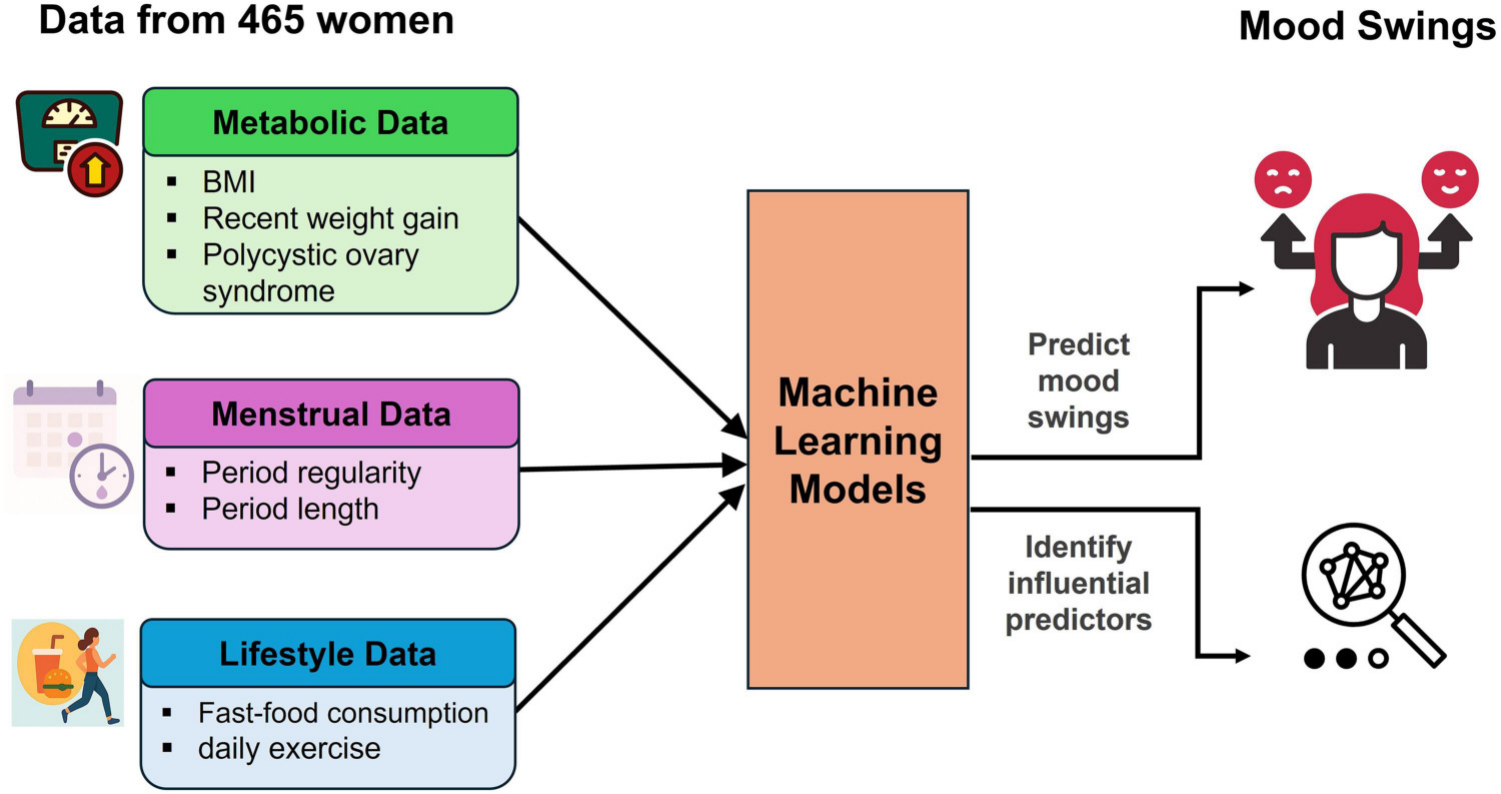
Study overview.

This study explores the use of ML models to predict mood swings in reproductive-age women using menstrual, metabolic, and lifestyle-related survey responses from a cohort of 465 women. A secondary objective is to identify the most influential predictors and their effects on the risk of mood swings (Figure 1). The central research question is whether self-reported menstrual, metabolic, and lifestyle features can be leveraged through ML to reliably identify women at heightened risk of mood instability. By addressing this question, the study aims to contribute evidence toward scalable, data-driven screening approaches in women's mental health, laying the groundwork for future integration of predictive models into preventive care and community-based interventions.

## 2 Methods

### 2.1 Dataset description

This study utilized a curated dataset comprising 465 responses from a survey targeting women of reproductive age. To align with the study's objective of examining metabolic, menstrual, and lifestyle influences on mood swings, the 15 collected features were grouped into conceptually relevant categories, as described in Table 1: metabolic indicators (weight, height, BMI, recent weight gain, PCOS diagnosis, excessive body/facial hair, skin darkening), menstrual indicators (period regularity, period length), lifestyle indicators (fast-food consumption, exercise daily), symptom burden (computed by summing the presence of five physical symptoms: recent weight gain, acne, hair loss, skin darkening, and facial hair), and demographic factor (age). The binary outcome variable was defined as self-reported experience of mood swings (Yes = 1, No = 0). The dataset exhibited class imbalance, with 354 positive cases (presence of mood swings) and 111 negative cases (absence of mood swings).

**Table 1:** Categories of features included in the analysis.

Category	Features	Rationale
Metabolic indicators	Weight, height, BMI, recently gained weight?, PCOS diagnosis, excessive body/facial hair, SKIN darkening	Reflect hormonal and metabolic health factors often implicated in mood variability.
Menstrual indicators	Menstrual period regularity, period length	Capture menstrual cycle regularity and duration, which may influence mood.
Lifestyle indicators	Fast food consumption, exercise daily	Represent modifiable lifestyle behaviors that may impact emotional well-being.
Symptom burden	Symptom_score, computed using weight gain, acne, hair loss, skin darkening, and facial hair	Capture cumulative self-reported physical and dermatological symptoms, some linked to hormonal disturbances, providing insight into overall symptom burden.
Demographics	Age	Accounts for life stage differences across reproductive-age women that may influence mood swings.

### 2.2 Data preprocessing

Categorical survey responses were recoded into numeric form: binary variables (e.g., PCOS diagnosis, recent weight gain, fast-food consumption, daily exercise, excessive body/facial hair, skin darkening, acne, hair loss) were encoded as 0/1 indicators, and menstrual regularity (regular vs. irregular) was similarly binarized.

Body mass index (BMI, kg/m²) was treated as a continuous feature alongside age, weight, height, and menstrual period length (days). The composite symptom burden score (symptom_score) was computed as the unweighted sum of five binary items (recent weight gain, acne, hair loss/thinning/baldness, skin darkening, and facial hair), producing an integer scale where higher values indicate greater cumulative symptom burden. Before model fitting, continuous features (age, weight, height, BMI, period length, and symptom_score) were standardized to zero mean and unit variance using parameters estimated from the training data; the same transformation was then applied to the test data to avoid information leakage.

Given the class imbalance (354 women with mood swings vs. 111 without), we applied the Synthetic Minority Over-sampling Technique (SMOTE) to the training set only, after the train–test split and scaling steps. SMOTE generates synthetic minority-class examples in feature space, thereby reducing imbalance while preserving the original test distribution.

### 2.3 Machine learning models

We trained five machine learning algorithms to predict the binary outcome of mood swings (Yes/No) from the 15 preprocessed survey features. The models were selected to cover a diverse set of non-linear classifiers, with an emphasis on tree-based ensemble methods and a margin-based kernel classifier that can capture complex interactions between metabolic, menstrual, and lifestyle predictors. The following models were used in our analysis:

▪ Random Forest: A bagging-based ensemble of decision trees that fits multiple trees on bootstrapped samples of the training data and aggregates their predictions by majority vote.
▪ Gradient Boosting: A sequential ensemble of shallow decision trees, where each tree is trained to correct the residual errors of the previous trees. By optimizing a differentiable loss function in a stage-wise manner, Gradient Boosting can model complex, non-linear decision boundaries while retaining control over overfitting through learning rate and tree-depth constraints.
▪ Light Gradient Boosting Machine (LightGBM): A gradient boosting framework that uses histogram-based decision tree learning and leaf-wise growth with depth constraints. LightGBM is designed for efficiency and scalability, enabling fast training and strong performance on structured tabular data with mixed feature types.
▪ Categorical Boosting (CatBoost): A gradient boosting algorithm that incorporates specialized handling of categorical variables via ordered target statistics and permutation-driven “ordered boosting,” which reduces target leakage and overfitting.
▪ Support Vector Machine (SVM) classifier: A maximum-margin classifier that seeks the hyperplane separating classes with the largest margin in feature space. Using a non-linear kernel, the SVM can project inputs into a high-dimensional space and learn flexible decision boundaries even when classes are not linearly separable in the original feature space.

### 2.4 Evaluation strategy

All models were evaluated using a stratified train–test split, with 25% of the data held out for testing to preserve the original class proportions. Within the training set, 5-fold cross-validation was used for model tuning and to obtain stable performance estimates. Performance was quantified using Accuracy, Area Under the Precision–Recall Curve (AUCPR), Precision, Recall, and F1-Score. This metric set captures both threshold-dependent (Accuracy, Precision, Recall, F1) and threshold-independent (AUCPR) aspects of discrimination, which is particularly important under the imbalanced distribution of mood-swing outcomes.

### 2.5 Feature importance analysis

We quantified feature contributions using two complementary approaches. Permutation Feature Importance (PFI) answers the question, “How much does the model's performance rely on this feature?” by measuring the decrease in accuracy when a feature is randomly permuted in the test set; larger drops indicate greater global importance. Shapley Additive exPlanations (SHAP) answers, “How does each feature push an individual prediction toward or away from mood swings, and by how much?” by attributing per-instance contributions whose magnitudes can be aggregated for a global ranking and whose signs provide directionality. Both methods were applied to the best-performing classifier under identical preprocessing and evaluated on the same held-out test set. For PFI, we used n_repeats = 30 to derive error bars. For SHAP, we employed the KernelExplainer, using a representative background from the training data.

## 3 Results

### 3.1 Cohort characteristics

**Figure 2:**
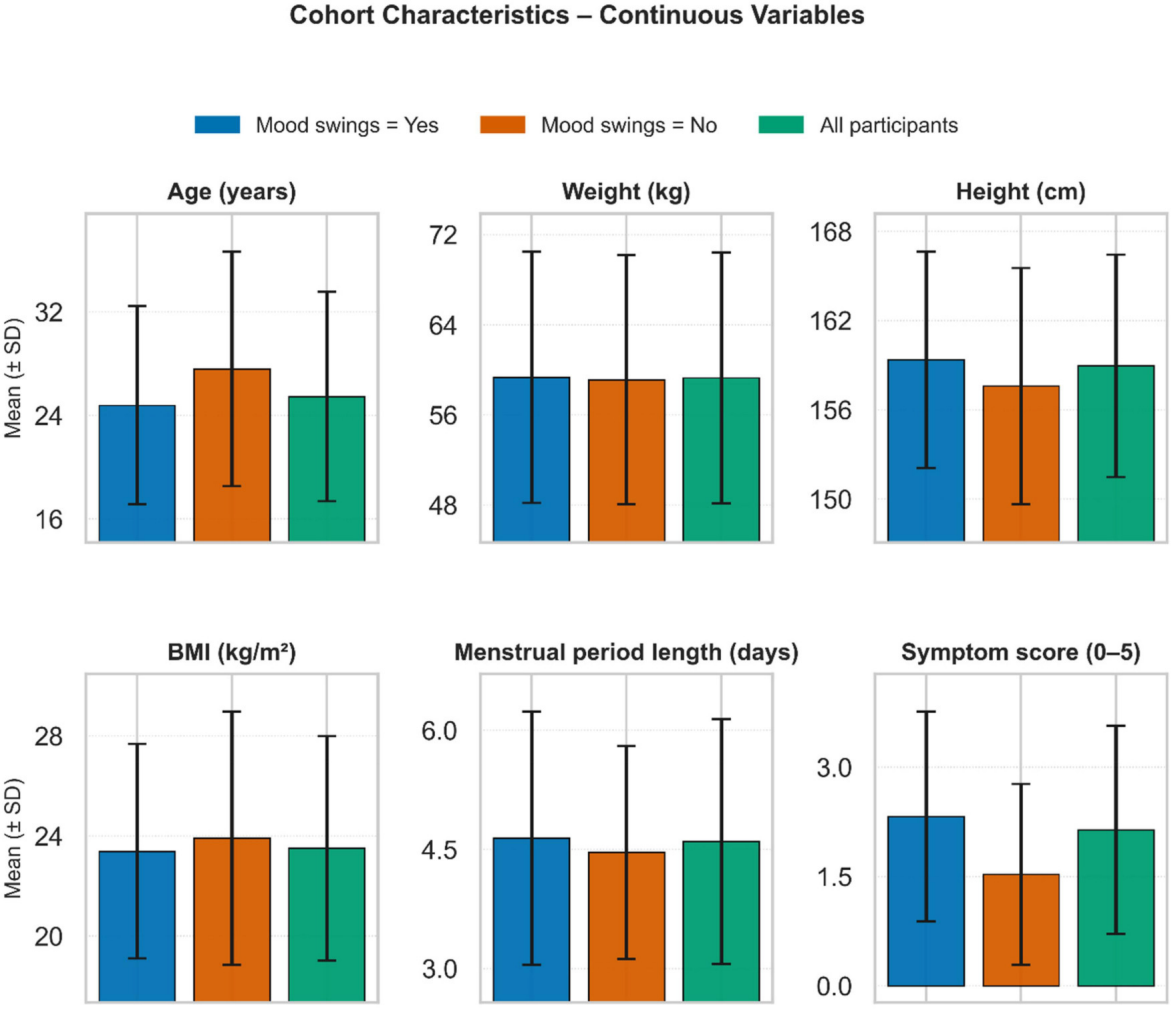
Cohort characteristics for continuous variables.

**Figure 3:**
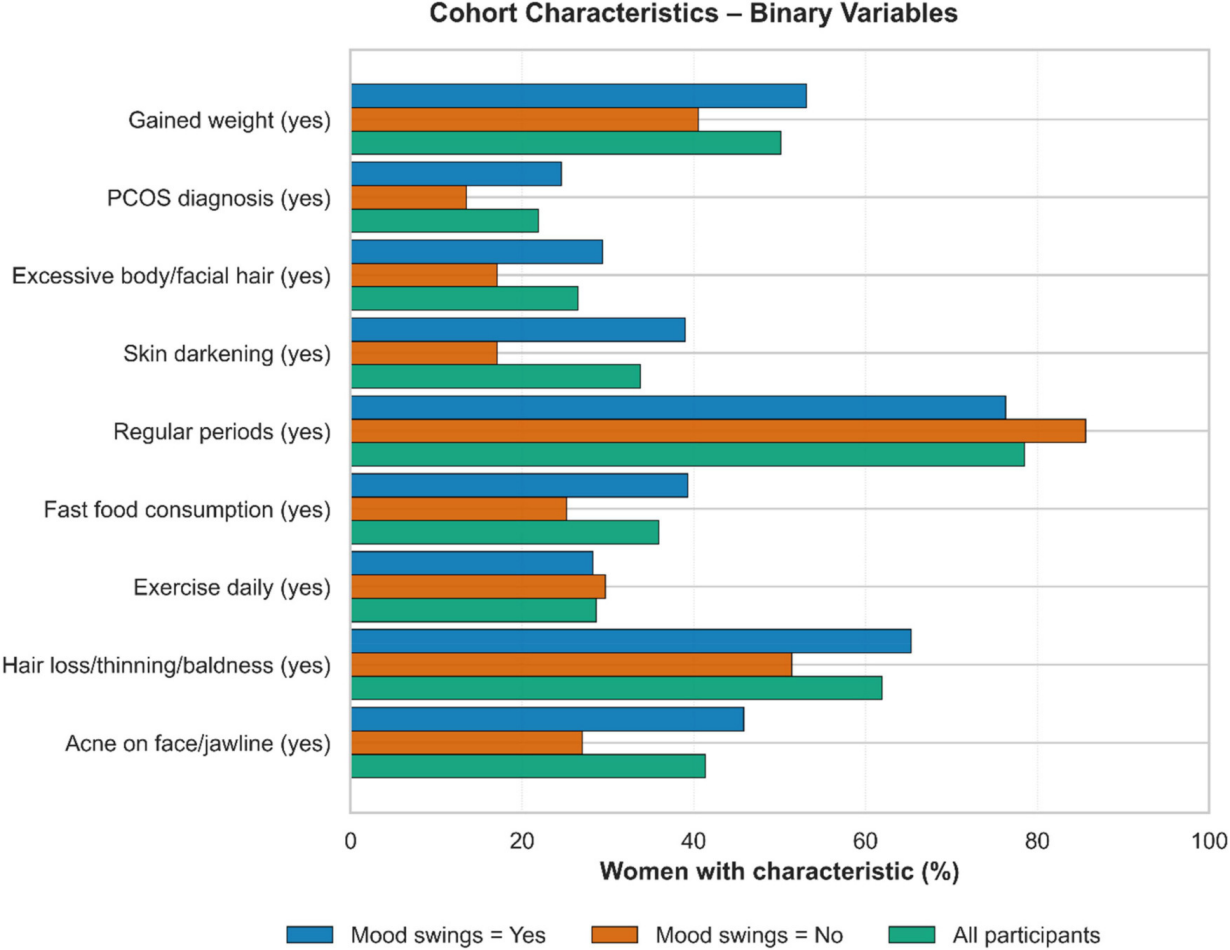
Cohort characteristics for binary variables.

The cohort comprised 465 women; 354 (76.1%) experienced mood swings and 111 (23.9%) reported not experiencing mood swings (Figures 2, 3). Overall, participants were young (mean age 25.46 ± 8.10 years), of normal weight (BMI 23.51 ± 4.48 kg/m²), and reported menstrual length of 4.60 ± 1.54 days; 78.5% had regular cycles. Compared with those without mood swings, the mood-swings group was younger (24.78 vs. 27.60 years) and had higher symptom burden (2.32 vs. 1.53). They more frequently reported recent weight gain (53.1% vs. 40.5%), PCOS diagnosis (24.6% vs. 13.5%), excessive body/facial hair (29.4% vs. 17.1%), skin darkening (39.0% vs. 17.1%), fast-food consumption (39.3% vs. 25.2%), hair loss (65.3% vs. 51.4%), and acne (45.8% vs. 27.0%).

### 3.2 Performance of machine learning models

**Figure 4:**
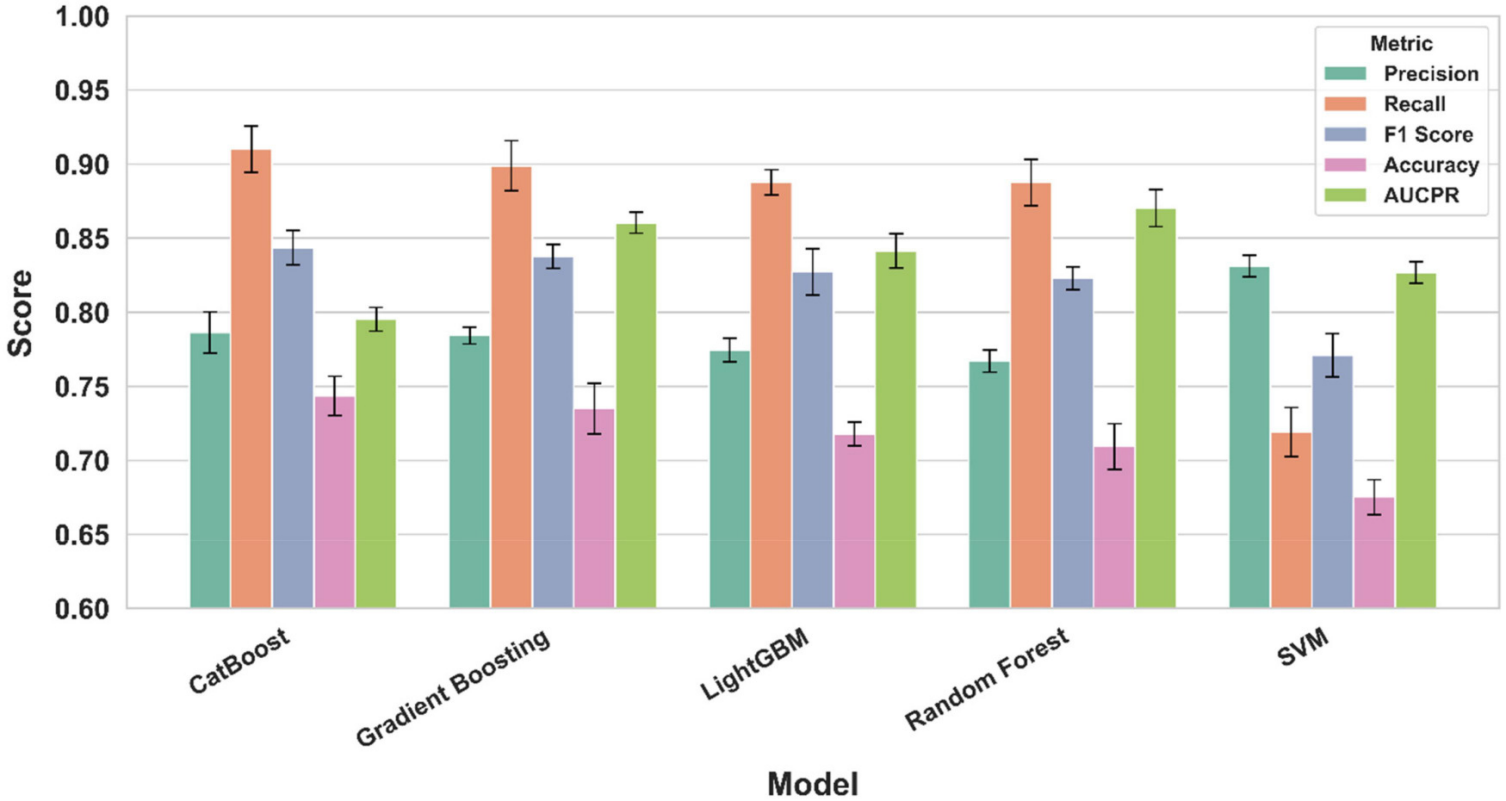
Comparison of evaluation metrics across models.

The performance of five supervised machine learning models was compared using multiple evaluation metrics (Figure 4). Error bars illustrate modest variability around each mean score and are largely overlapping across models, indicating that differences in performance are relatively small. CatBoost achieved the highest recall (0.91) and F1 score (0.84), while Gradient Boosting (recall 0.90, precision 0.78) and LightGBM (recall 0.89, precision 0.77) also performed strongly across recall and precision. Random Forest yielded the highest AUCPR (0.87), indicating superior precision–recall balance, whereas Support Vector Machine demonstrated the strongest precision (0.83) but lower recall (0.72) and F1 score (0.77). Accuracy values ranged from 0.68 (SVM) to 0.74 (CatBoost), underscoring comparable overall classification ability.

### 3.3 Feature importance analysis

#### 3.3.1 Permutation feature importance analysis

**Figure 5:**
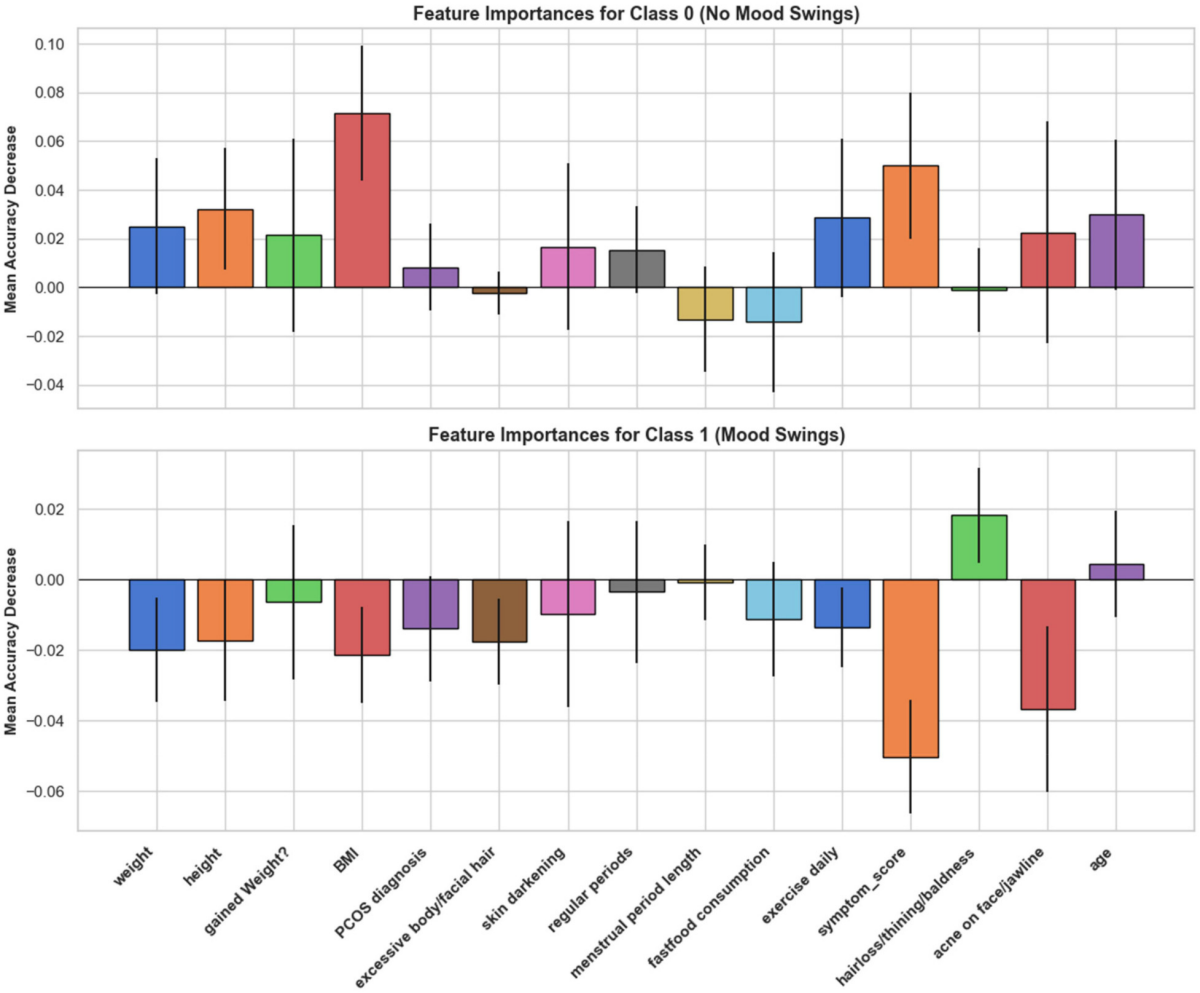
Class-Specific permutation feature importance (random forest model).

Figure 5 shows class-conditional permutation importance for the best-performing classifier. Importances are reported as the mean decrease in accuracy after shuffling each feature; larger-magnitude drops indicate stronger contribution to prediction.

**Figure 6:**
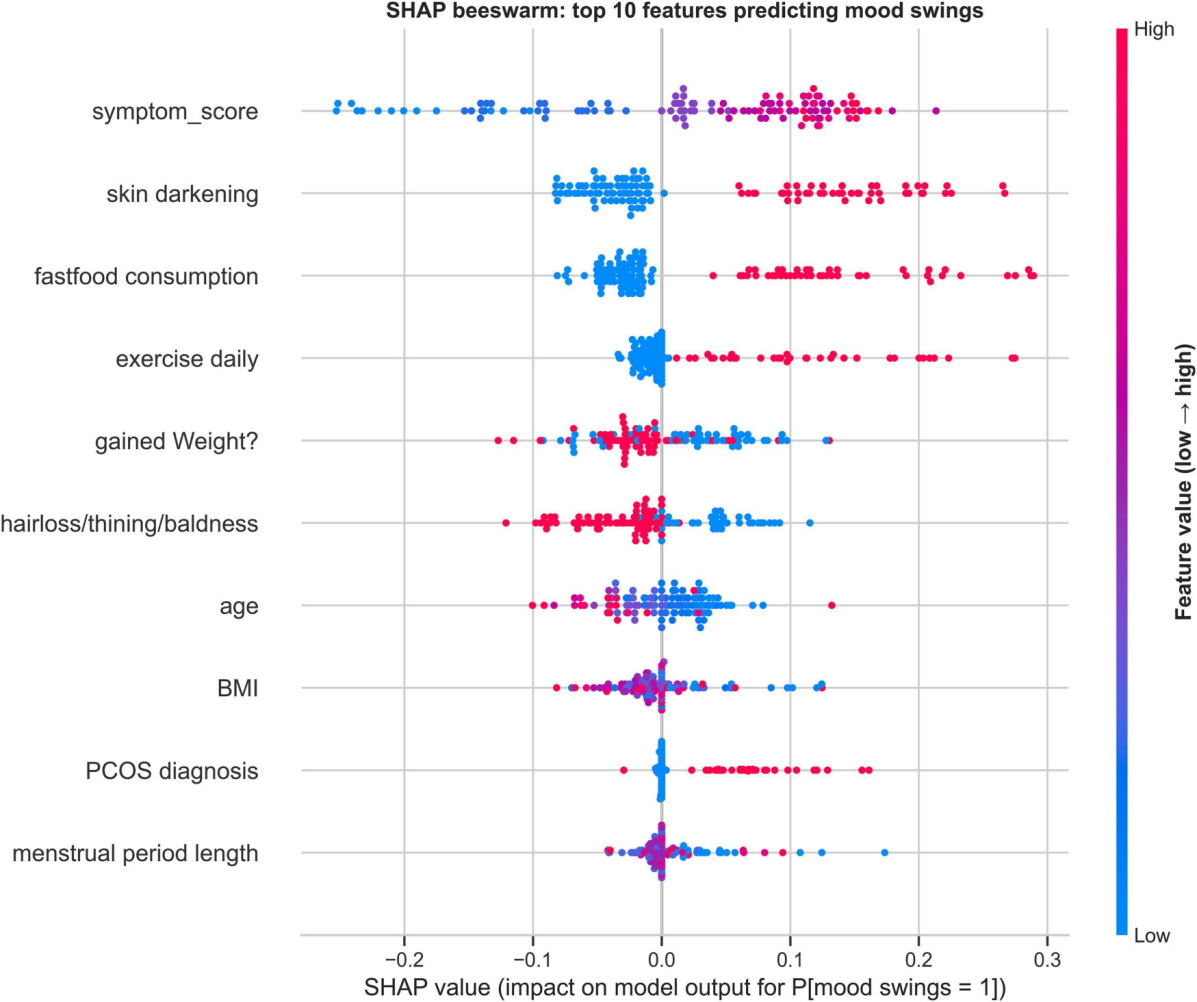
SHAP beeswarm (top 10 features) for predicting mood swings (random forest model). Features are ordered by global importance (mean absolute SHAP value). Each dot represents one participant; the horizontal position is the SHAP value [feature's impact on the model output for P(mood swings = 1)]. Values to the right increase, and values to the left decrease, the predicted probability of mood swings. Dot color encodes the original feature value (red = higher, blue = lower).

For Class 0 (no mood swings), performance was most sensitive to anthropometric features. BMI produced the largest accuracy decrease, with additional contributions from recent weight gain and age. Menstrual indicators (menstrual period length, regular periods) and dermatologic variables (hair loss, acne, skin darkening) exerted smaller effects, and most remaining features clustered near zero.

In Class 1 (mood swings), the dominant driver was symptom_score, followed by gained weight, BMI, hair loss/thinning/baldness, and skin darkening. By contrast, regular periods and menstrual period length were close to null, and PCOS diagnosis contributed little additional information.

Taken together, per-class PFI suggests that absence of mood swings is best characterized by basic anthropometrics and age, whereas presence of mood swings is driven chiefly by symptom burden with supporting signals from metabolic/dermatologic markers.

#### 3.3.2 SHAP feature importance analysis

SHAP global explanations indicate that variables reflecting symptom burden and metabolic/dermatologic status dominate prediction of mood swings (Figure 6). Higher symptom_score showed the largest positive SHAP values, strongly increasing the model-estimated probability of mood swings. Elevated skin darkening and fast-food consumption also pushed predictions toward the positive class, as did recent weight gain and a PCOS diagnosis (moderate effects). Age displayed a negative direction—younger participants (lower values, blue) tended to increase risk—while BMI showed a modest inverse pattern (higher BMI slightly lowering risk). Exercise daily and hair loss/thinning/baldness had smaller, mixed contributions, and menstrual period length exerted only minor negative influence. Overall, SHAP highlights symptom burden plus lifestyle and dermatologic markers as key drivers.

## 4 Discussion

### 4.1 Main findings

Using survey-derived metabolic, menstrual, and lifestyle features, machine-learning models predicted self-reported mood swings with good overall performance. CatBoost, Gradient Boosting, and LightGBM achieved the strongest balance of recall (up to 0.91) and F1 score (up to 0.84), highlighting their ability to identify women at risk of mood swings with high sensitivity and overall performance. Random Forest yielded the highest AUCPR (0.87), indicating robust precision–recall trade-offs, while SVM showed the best precision (0.83). Collectively, these results demonstrate that ensemble-based methods provide consistent predictive value across evaluation metrics. Interpretability analyses (PFI, SHAP) converged on symptom burden as the dominant predictor, with additional contributions from lifestyle factors, specifically fast-food consumption and daily exercise, whereas menstrual regularity/length contributed minimally. These results support pragmatic screening using a small, high-yield feature set.

### 4.2 Agreements and discrepancies in feature importance analysis

Both feature-importance analyses, PFI and SHAP, converged on a core signal aligned with the study objective. Symptom burden was consistently the strongest driver of mood-swing risk: SHAP showed large positive contributions of symptom_score, and per-class PFI identified it as the dominant feature for Class 1. Dermatologic/metabolic markers, notably skin darkening and recent weight gain, also increased risk in both analyses. Conversely, menstrual indicators (regular periods, menstrual length) were near-null across methods. PFI further indicated that the absence of mood swings (Class 0) is best characterized by anthropometrics and age, a pattern compatible with SHAP's negative direction for age and modest influence of BMI.

Some divergences are informative. BMI was highly important for Class 0 under PFI but showed only modest, slightly inverse effects in SHAP; fast-food consumption appeared salient in SHAP but was minor in PFI; and PCOS diagnosis had limited PFI impact yet moderate positive SHAP effects. These differences likely reflect (i) what is measured—PFI quantifies metric-linked performance drops after independent shuffling, whereas SHAP attributes directional, local contributions; (ii) correlations among features (e.g., BMI, weight, weight gain) that can dilute PFI; (iii) class-specific evaluation for PFI vs. global aggregation for SHAP; and (iv) variance from smaller class-conditioned test subsets.

Taken together, PFI provides a metric-anchored global ranking, while SHAP shows how features push predictions across individuals. Combining them strengthens inference: mood swings are chiefly explained by symptom burden with supportive signals from metabolic/dermatologic features, anthropometrics help define low-risk profiles, and menstrual variables contribute little.

### 4.3 Clinical and research implications

The pattern of feature importance observed in our models suggests plausible psychobiological pathways linking metabolic and lifestyle disturbances to mood swings. Symptom burden and dermatologic markers such as skin darkening likely index underlying insulin resistance and hyperandrogenism, which can disrupt hypothalamic–pituitary–ovarian signaling, alter cortisol dynamics, and affect monoaminergic neurotransmission implicated in mood regulation (26–28). Concurrently, recent weight gain and higher fast-food consumption may exacerbate adiposity, glycemic variability, and systemic inflammation, while lower exercise opportunities reduce neurotrophic and anti-inflammatory benefits of physical activity, together amplifying vulnerability to mood disturbances (29). The limited contribution of menstrual cycle metrics in our models suggests that, in this cohort, chronic metabolic and lifestyle factors may be more salient drivers of mood swings than cyclical hormonal variation alone, aligning with an integrated reproductive–metabolic–behavioral framework.

Clinically, the prominence of symptom burden (higher symptom_score)—computed from five easily assessed physical symptoms (weight gain, acne, hair loss, skin darkening, and facial hair)—suggests that brief symptom checklists could function as efficient front-end screeners for mood-swing risk in reproductive-age women, prompting timely psychosocial assessment and lifestyle counseling. The fact that components such as skin darkening and recent weight gain also emerged as important individual predictors supports the practical use of both a composite checklist and salient single findings in routine care. At the low-risk end, age and basic anthropometrics may help identify women unlikely to require intensive follow-up, supporting more efficient allocation of behavioral-health resources. Taken together, these patterns could inform a pragmatic risk tool integrating symptom burden with key metabolic and dermatologic indicators, subject to prospective evaluation, external validation, and calibration before clinical use.

From a research standpoint, our findings motivate several methodological and translational directions. First, quantify clinical utility via calibration and decision-curve analysis (9). Second, develop dynamic, individualized prediction–and–intervention pipelines that fuse wearable/smartphone streams (sleep, HRV, activity, circadian regularity) with ecological momentary assessment and large language model (LLM)-enabled summarization/coaching to forecast the timing and intensity of mood swings and deliver just-in-time prompts; evaluate time-varying models (state-space/transformers) and prospective studies for safety, fidelity, and impact. Third, broaden inputs and mechanisms—menstrual tracking, medications, diet, stress—and test links among dermatologic markers, insulin resistance, and neuroendocrine pathways. Fourth, ensure generalizability and equity via external, multi-site validation, subgroup fairness audits (age, BMI strata, PCOS status), and uncertainty quantification (calibrated probabilities, conformal risk bands).

### 4.4 Study limitations

This study has several limitations. First, outcome and predictors are self-reported, introducing recall and reporting bias; the outcome is not a clinical diagnosis. Second, the cross-sectional survey design precludes inference about temporal ordering between exposures and mood swings. Third, the cohort (*n* = 465) may reflect selection bias and limits generalizability beyond similar populations; external validation and recalibration are required. Fourth, several potentially important variables were unavailable (e.g., sleep duration/quality, medication use, psychosocial stress), which may attenuate performance and bias feature attribution.

## 5 Conclusion

Machine learning models applied to low-cost, survey-derived features predicted mood swings in reproductive-age women with good performance. Across methods, symptom burden emerged as the dominant driver, with additional contributions from lifestyle and metabolic/dermatologic indicators, while menstrual regularity/length contributed minimally. These findings support pragmatic, data-light screening strategies and hypothesis generation about metabolic-mood links. Translation to practice will require external validation and calibration, as well as prospective studies, potentially integrating wearables and EMA to evaluate clinical utility, generalizability, and the impact of targeted interventions.

## Data Availability

Publicly available datasets were analyzed in this study. This data can be found here: https://www.kaggle.com/datasets/sahilkoli04/pcos2023/data.
